# A novel bilateral anterior sacrospinous hysteropexy technique for apical pelvic organ prolapse repair via the vaginal route: a cohort study

**DOI:** 10.1007/s00404-022-06486-4

**Published:** 2022-03-14

**Authors:** Gert Naumann, Clara Börner, Lena-Johanna Naumann, Sebastian Schröder, Tanja Hüsch

**Affiliations:** 1 Department of Gynecology and Obstetrics, Helios Hospital Erfurt, Nordhäuser Straße 74 99089, Erfurt, Germany; 2 Department of Gynecology and Obstetrics, University Hospital Düsseldorf, Düsseldorf, Germany; 3 Department of Gynecology and Obstetrics, Medical Center University Lübeck, Ratzeburger Allee 160, 23538 Lübeck, Germany; 4 Department of Gynecology and Obstetrics, KMG Hospital Sondershausen, Sondershausen, Germany; 5 Department of Urology and Pediatric Urology, University Medical Center of Johannes Gutenberg University, Mainz, Germany

**Keywords:** Uterine prolapse, Pelvic floor disorders, Pelvic organ prolapse, Surgical mesh, Gynaecologic surgical procedures, Therapy

## Abstract

**Background:**

Uterine-preserving techniques are becoming increasingly popular in the last decade. This investigation evaluates a novel hysteropexy technique using a mesh in sling-alike configuration [Splentis (Promedon, Argentina)] which is attached anteriorly to the cervix and suspended to the sacrospinous ligaments bilaterally via the vaginal route in women undergoing surgery for uterine prolapse.

**Methods:**

This was a single-center cohort study, evaluating women who underwent transvaginal hysteropexy with Splentis for primary uterine descent. Data have been collected prospectively as part of the quality assurance system. Primary endpoint was treatment success, defined as a combined endpoint including the absence of a vaginal bulge symptom and no retreatment of apical prolapse. A validated questionnaire to evaluate quality-of-life and prolapse symptoms was utilized. Descriptive analysis was applied. Wilcoxon signed-rank test was performed to compare paired samples. The significance level was set at 5%.

**Results:**

A total of 103 women with a median age of 68.0 [IQR 11.5] years with a median apical POP-Q stage of 3 were included. The median surgery time was 22 [IQR 12] minutes and no intraoperative complication occurred. After a median follow-up time of 17 months, treatment success was achieved in 91 (89.2%) patients and quality of life and patient report outcomes improved significantly (*p* < 0.001). Mesh exposure occurred in 3 (2.9%) patients. Of these, two patients required surgical revision, and one patient was treated conservatively. One patient required partial mesh removal due to dyspareunia.

**Conclusion:**

Bilateral sacrospinous hysteropexy with Splentis offers an efficacious and safe alternative for apical compartment repair, incorporating the advantages of pelvic floor reconstruction via the vaginal route.

**Supplementary Information:**

The online version contains supplementary material available at 10.1007/s00404-022-06486-4.

## Background

Pelvic organ prolapse (POP) is defined as a downward descent of the pelvic organs that results in protrusion of the vagina, uterus, or both [1]. It is a common condition, with a prevalence of approximately 30–40% [2] and a lifetime risk of undergoing surgery for POP of 10–20% [3]. Although the uterus plays a passive role in the development of POP and hysterectomy does not necessarily correct the underlying defect in the apical vaginal support structures [4], hysterectomy was formerly most commonly performed during POP repair [5]. However, there is an increasing trend in uterine-preserving surgical techniques [6]. Clinical investigations have demonstrated that 36–60% of women prefer preservation of the uterus if surgical repair for symptomatic apical prolapse is required [7]. Reasons for uterine preservation include the belief of the uterus and ovaries have an impact on sexual function and activity or sense of identity and the surgical risks of hysterectomy itself [7]. The surgical risks of hysterectomy include, among others, prolonged surgery, increased blood loss, and the occurrence of enteroceles [7, 8].

There are several hysteropexy techniques that may be differentiated in regard to the route of surgical access (vaginal vs. transabdominal) and the utilization of sutures or a mesh for uterine fixation [4]. Splentis (Promedon, Cordoba, Argentina) is a lightweight, type I polypropylene mesh used in uterine-preserving techniques via the vaginal route for women with apical POP. It is designed in a sling-alike configuration; the mesh is fixed anteriorly to the cervix and suspended to the sacrospinous ligaments (SSLs) bilaterally. Importantly, in comparison to other transvaginal implanted meshes, Splentis is neither attached to, nor it is supporting the anterior vaginal wall directly. The indication for Splentis is uterine descent. The theoretical benefits include preservation of the physiological axis of the vagina. Furthermore, direct fixation to the vaginal wall is avoided, which might preserve mobility of the vagina and thus normal pelvic floor function.

The present study was performed to evaluate the efficacy and safety of vaginal sacrospinous hysteropexy using Splentis in women undergoing primary surgery for uterine descent.

## Methods

This was a single-center, single-arm cohort in a community maximum care hospital. The study center is a certified pelvic organ prolapse center and data have been collected prospectively as part of the quality assurance system. The protocol was approved by the ethics committee of the medical association of Thuringia, Germany (vote number: 60750/2020/26), informed consent was obtained by the patients and the trial has been registered at the German Clincial Trials Register (number: DRKS00018990) prior to patient recruitment. The investigation was performed in accordance with the principles of Good Clinical Practice and the current version of the declaration of Helsinki; international and national regulations were complied with. The manuscript was prepared in accordance with the STROBE guideline.

Non-fertile women who underwent primary transvaginal POP repair using Splentis (Promedon, Cordoba, Argentina) for uterine descent between 2017 and 2019 with a minimum follow-up time of 12 months were invited to give consent for data analysis. Non-fertile women were defined as women in menopause or permanently unable to become pregnant due to iatrogenic causes. Surgeons implanting Splentis were experienced in transvaginal pelvic floor reconstruction. The indication for hysteropexy with Splentis were non-fertile women with a symptomatic apical POP-Q ≥ 2 and the absence of indication for hysterectomy. Importantly, Splentis implantation was not combined with other mesh procedures such as sling surgery for stress urinary incontinence. Women received perioperatively local estrogen therapy if not contraindicated. Patients were introduced to avoid heavy lifting, excessive physical exercise and sexual intercourse or other vaginal insertions for at least six weeks after surgery.

The primary endpoint was treatment success. Treatment success was defined as a combined endpoint [9] including the patient reported outcome of absence of a vaginal bulge symptom and no need for surgical or conservative retreatment for apical POP. Emphasizing herby the importance of subjective assessment in POP surgery which has been put into focus in the recommendation of outcome success evaluation for POP surgery [9, 10]. Therefore, this certified study center evaluates systematically treatment success by the utilisation of a validated questionnaire and interview follow-up after 12 months as part of the quality assurance system. Secondary outcomes included the number of adverse events, number of further surgeries required for complications, quality of life (QoL) and estimated exposure-free and anatomical failure-free survival rates.

Demographic information, the results of the perioperative course and any unscheduled follow-up were collected from the medical records. The operative duration was defined as the time from the first incision to the end of wound closure. Complications were reported according to the Clavien–Dindo classification.

The results of a prospectively collected telephone interview and validated questionnaire, which is performed as part of the quality assessment of the study site 12 months after surgery, were included. The interview comprised by the questions regarding subjective treatment success, overall satisfaction with the surgery, the presence of vaginal bulge symptom, the presence of a palpable or visible vaginal bulge, sexual activity and dyspareunia, the occurrence of any adverse events or further surgeries since the last clinical follow-up, the utilization of a pessary, change in prolapse-related symptoms (from very much better to very much worst), pain and the presence and type of urinary incontinence. QoL and prolapse-related symptoms were assessed according to the German Pelvic Organ Prolapse Questionnaire (POP-Q) at baseline and at interview follow-up. The POP-Q is a validated, standardized QoL questionnaire for women with POP, including four domains (bowel, urinary, sexual, and prolapse symptoms) with scores ranging from 0 to 10 (a higher score indicates a more negative impact), as well as a total score (range, 0–40) combining the results of all domains. Pain was assessed postoperatively and at the follow-up interview using the verbal analogue scale (VAS/VRS) (range, 0–10; a higher score indicates a more negative impact).

### Surgical technique

Hydrodissection and full-thickness vaginal wall dissection have been performed. Then, the vesicovaginal and subsequently the pararectal space were developed by blunt and sharp dissection, as appropriate. The ischial spines and SSLs were identified by palpation. The tissue surrounding the SSL was carefully moved away from the ischial spine along the ligament using the index finger. The tissue anchoring system (TAS) consists of single anchors attached to nonabsorbable monofilamentous sutures. The anchor was fixed to the SSL by single-use instruments bilaterally. Then, the central part of the mesh was placed on the anterior supravaginal portion of the cervix, and the mesh was attached with three nonabsorbable sutures. Subsequently, each end of the mesh sling was fixed to the corresponding SSL by knotting the corresponding sutures of the TAS. Additionally, anterior colporrhaphy with plication of the anterior endopelvic fascia was performed with running polydioxanone sutures. Wound closure was performed according to the surgeon’s preference, followed by vaginal packing for 24 h.

### Statistical analysis

Descriptive data are presented as the median [interquartile range] or mean (standard deviation). Categorical variables are presented as numbers and frequencies. Time-dependent variables are presented using Kaplan–Meier curves. Univariate and multivariate analyses were performed to identify variables that predict the primary outcome. The following variables were utilized to identify risk factors for treatment failure: age, body mass index, POP-Q classification, and number of births. Differences between groups were determined by the Mann–Whitney *U* test, Fisher’s exact test or log-rank test, as appropriate. McNemar or Wilcoxon signed-rank test was performed to compare paired samples. Quantitative variables were not grouped. The number of participants reflects the total number of surgeries and the total number of patients who provided consent to participate in the trial.

Please change in: In the case of missing POP-Q classification, sensitivity analyses were applied for estimated survival according Kaplan–Meier according to all subjects with missing data were treated successfully according to the results of the phone interview. A significance level of 5% was considered to be statistically significant. Statistical analysis was performed using R version 4.0.2.

## Results

A total of 103 women with a median age of 68.0 [IQR 11.5] years were included. The median anterior and apical POP-Q stage was 3 at baseline. The complete list of baseline characteristics is presented in Table 1.

**Table 1:** Baseline characteristics

Variable	*n* = 103
Age in years, median [IQR]	68.0 (11.5)
Body mass index (kg/m^2^), median [IQR])	26.0 (2.9)
Postmenopausal status, *n* (%)	103 (100)
Number of child births, *n* (%)	
0	4 (3.9)
1	16 (15.7)
2	66 (64.7)
3	13 (12.7)
≥ 4	3 (2.9)
Residual urine (ml), mean (SD)	9.04 (35.3)
Residual urine > 100 ml, *n* (%)	6 (5.8%)
No sexual activity, *n* (%)	45 (44.1)
Due to dyspareunia, *n* (%)	6 (5.8)
Missing Partner, *n* (%)	24 (23.3)
No desire/libido, *n* (%)	15 (14.6)
POP-Q staging	
Anterior vaginal wall, *n* (%)	
1	5 (4.9)
3	41 (39.8)
4	57 (55.3)
Apical vaginal wall, *n* (%)	
2	29 (28.2)
3	68 (66.0)
4	6 (5.8)
Posterior vaginal wall, *n* (%)	
0	11 (10.7)
1	87 (84.5)
2	3 (2.9)
3	2 (1.9)

*POP* pelvic organ prolapse, *POP*-*Q* pelvic organ prolapse quantification, *COPD* chronic obstructive pulmonary disease

The median surgery time was 22 [IQR 12] minutes, and no blood loss > 200 ml occurred. Additional anterior colporrhaphy was performed in 102 (99.0%) patients, and posterior colporrhaphy was performed in 4 (3.9%) patients. There were no intraoperative complications, particularly no cases of injury to surrounding vessels or organs. The mean postoperative pain score was 0.7 (SD 0.9). There were no postoperative complications except for two (1.9%) cases of residual urine > 100 ml; these patients required either intermittent self-catheterization or pharmacotherapy with myocholine. The residual urine was completely resolved in these women at follow-up.

### Follow-up

The results of 102 (99.0%) telephone interviews and questionnaires were available at follow-up. One (1.0%) woman died due to myasthenia gravis exacerbation independently from Splentis implantation. The median follow-up time was 17 [IQR 4] months.

The absence of a vaginal bulge symptom was reported by 91 (89.2%) patients and no patient required repeat surgery due to prolapse recurrence, indicating a treatment success in 91 (89.2%) patients.

**Fig. 1:**
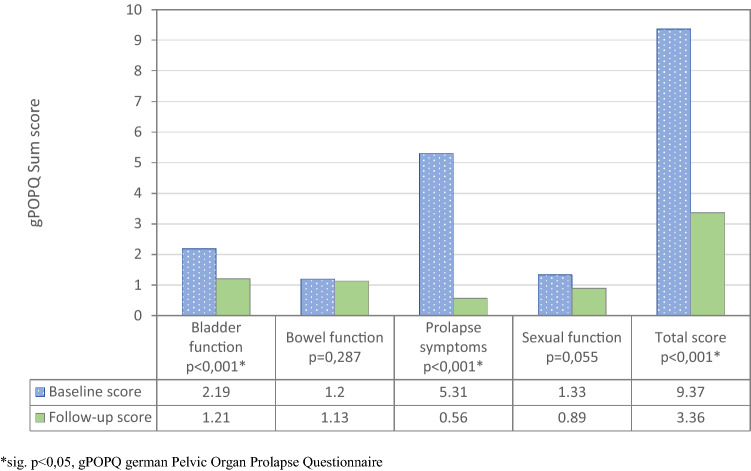
POP-Q domains compared between baseline and follow-up

**Fig. 2:**
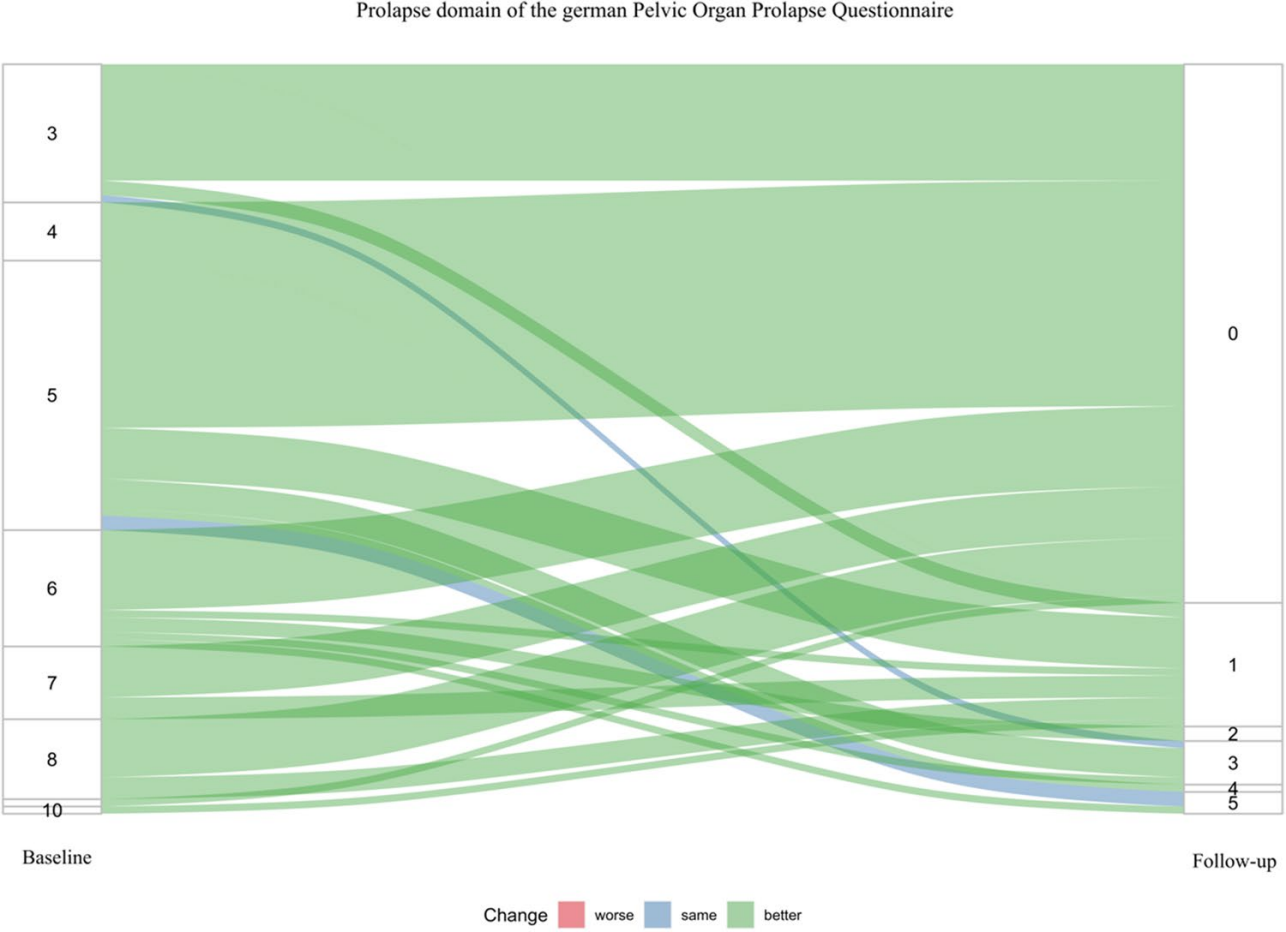
Prolapse symptoms in comparison between baseline and follow-up according to the German Pelvic Organ Prolapse Questionnaire

A total of 99 (97.1%) patients reported subjective treatment success. QoL and prolapse-related symptoms improved significantly at follow-up compared with baseline according the POP-Q (Fig. 1). In particular, significant improvement of prolapse related symptoms was reported in comparison between baseline and follow-up (*p* < 0.001, Fig. 2). The mean pain score decreased from 0.7 (SD 0.9) postoperatively to 0.3 (SD 1.0) at follow-up. The complete list of results is presented in Table 2.

**Table 2:** Results of interview follow-up

Variable	*n* = 102
Subjective treatment success, *n* (%)	99 (97.1)
Satisfaction with surgery, *n* (%)	94 (92.2)
Prolapse related symptoms, *n* (%)	
Very much better	83 (81.4)
A little better	16 (15.7)
Unchanged	3 (82.6)
Worse	0
Vaginal bulge symptom, *n* (%)	11 (10.8)
Visible or palpable vaginal bulge, *n* (%)	6 (5.9)
No sexual activity, *n* (%)	54 (52.9)
Dyspareunia, *n* (%)	2 (1.8)
Further surgery due to dyspareunia and granulation polyp, *n* (%)	1 (1.0)
Repeated surgery for prolapse recurrence, *n* (%)	0
Further surgery for adverse events since last follow-up, *n* (%)	0
Pessary utilization due to apical or anterior recurrent prolapse, *n* (%)	0
VAS of pain, mean (SD)	0.326 (0.956)
Stress urinary incontinence, *n* (%)	16 (15.7)
deNovo, *n* (%)	8 (7.8)
Occult SUI at Baseline, *n* (%)	1 (1.0)
Persistent, *n* (%)	7 (6.9)

*VAS* visual analog scale, *SD* standard deviation

Mesh exposure occurred in three (2.9%) patients. One (1.4%) patient did not require surgical treatment (size: 3 mm), and exposure resolved completely by conservative treatment. Two (1.9%) patients required further surgery by either wound closure (size: 15 mm) or partial mesh resection and wound closure (size: 5 mm).

One (0.9%) patient received a midurethral sling due to persistent stress urinary incontinence (SUI) three months after Splentis implantation. SUI was already present at baseline accompanied by impairment of health-related QoL according to the POP-Q. SUI resolved completely after midurethral sling surgery, and health-related QoL improved accordingly.

Dyspareunia was reported by six (5.8%) patients at baseline which resolved after surgery. Of two (1.8%) patients with deNovo dyspareunia, one (1.0%) underwent partial mesh removal due to dyspareunia and the development of granulation tissue at the time of the follow-up interview. The domain sexual function of the POP-Q did not demonstrate a statistically significant difference between baseline and follow-up (*p* = 0.055).

A total of 16 (15.7%) patients reported SUI at the follow-up interview, of whom eight (7.8%) had de novo SUI. None of these patients reported a history of or planned surgery to treat SUI at the last follow-up. Overall complication rates classified by Clavien–Dindo are presented in Table 3.

**Table 3:** Complication rates at clinical and interview follow-up classified by Clavien–Dindo

Variable	Clavien–Dindo					
	NA	I	II	III		IV
				a	b	
Bacterial or mycotic vaginosis, *n* (%)	0					
Clinical infection of the study device, *n* (%)	0					
Impaired wound healing, *n* (%)	0					
Mesh exposure or extrusion, *n* (%)		1 (1.0)			2 (1.9)	
Symptomatic residual urine or urinary retention, *n* (%)	0					
Dyspareunia, *n* (%)		1 (1.0)			1 (1.0)	
Stress urinary incontinence, *n* (%)		15 (14.6)			1 (1.0)	
Contraction of the study device, *n* (%)	0					
Dehiscence, *n* (%)	0					
Folding of the mesh, *n* (%)	0					
Repeated surgery for prolapse recurrence, *n* (%)	0					
Residual urine > 100 ml, n (%)	0					

## Discussion

Treatment success of apical compartment prolapse with bilateral anterior sacrospinous hysteropexy using Splentis was 89.2% after a median follow-up of 17 months. Additionally, QoL and prolapse-related symptoms improved significantly after surgery. There were three (2.9%) patients with mesh exposure of which two (1.9%) required revision surgery and one (1.4%) was treated conservatively. One (1.4%) patient required further surgery due to dyspareunia and the formation of a granulation polyp. No patient required repeat surgery or a pessary due to recurrence of an apical or anterior prolapse.

In comparison to traditional posterior sacrospinous fixation, SSL suspension with Splentis is performed using single-use instruments to place an anchor with attached sutures to each SSL. Uterine suspension is facilitated by placing a mesh in a sling-like configuration anteriorly to the cervix and suspended to the SSL bilaterally using the sutures attached to the anchors. In comparison, there are several hysteropexy techniques that may be differentiated in regard to the surgical route (vaginal vs. abdominal) and the utilization of native tissue or synthetic mesh for uterine fixation [4]. Formerly, several synthetic meshes were available for the use via the vaginal route and indicated for apical POP. These meshes had a larger surface area and were attached to the anterior vaginal wall to address the apical and anterior compartments simultaneously. However, the surface size and the attachment of mesh directly to the vaginal wall has been identified as crucial risk factors for mesh-related morbidity [11] which led to a discreditation of these meshes [12]. Furthermore, apical POP repair by sacrocolpopexy or hysteropexy techniques still often include the utilization of a mesh and these techniques have not been affected by the FDA mesh ban due to the lower risk of mesh-related morbidity. In contrast, Splentis is not attached to the vaginal wall, the mesh surface is significantly smaller and the indication is limited to apical compartment prolapse. Therefore, Splentis does not fall into the scope of mesh-augmented anterior POP repair and the FDA mesh ban.

However, although the use of transvaginal synthetic meshes for POP repair has been extensively discussed by the FDA and these products have been removed from the US market in 2019 [4], international medical and scientific associations, such as the Scientific Committee on Emerging and Newly Identified Health Risks (SCENIHR) [5], the European Association of Urology (EAU) and the European Urogynaecological Association (EUGA) [6] the Gynecology and American Urogynecologic Society (AUGS) [7] and the International Federation of Gynecology and Obstetrics (FIGO) [8] clearly state that synthetic meshes are still an important treatment option for POP repair. In fact, it is a safe treatment recommended for patients with specific clinical characteristics if the procedure is performed by experienced surgeons and mesh material with certain characteristics is utilized. Thus, under these conditions, it can be concluded that the risks are outweighed by the benefits. Nevertheless, these statements are targeting meshes which where directly attached to the vaginal wall.

Additionally, the SCENIHR states that the outcome of mesh in POP repair depend on the material properties, product design, overall mesh size, route of implantation, patient characteristics, associated procedures (such as hysterectomy) and surgeons experience. Several aspects have been considered in the design of Splentis to reduce complication rates including material properties, reduced overall mesh size and avoidance of direct contact of the mesh to the vaginal wall. Furthermore, the surgeon in this investigation is highly experienced in pelvic floor reconstruction. Finally, mesh-augmented repair is still widely accepted and used in surgical AP repair in procedures such as sacrohysteropexy and sacrocolpopexy, the latter being considered the current state of the art in POP treatment.

Treatment success was achieved in 89.2% patients in the current investigation. No patient required repeat surgery, which is consistent with the results of other hysteropexy techniques using mesh or sutures via the abdominal or vaginal route. The overall objective treatment success rate of apical compartment repair, including any kind of transabdominal laparoscopic hysteropexy technique, was 85.3% according to a recent meta-analysis [13]. The rate of subjective treatment success has been reported range from 73 to 10%, with repeat surgery performed in 0–28% of patients [13]. Taking into consideration only studies that used a synthetic mesh for uterine suspension via the abdominal route, the pooled success rate was 92% [14]. Considering any native tissue repair performed via the vaginal route, the rate of treatment success in the apical compartment was reported to range from 70.2 to 89.8% [15, 16]. In particular, the reported success rate for transvaginal sacrospinous fixation ranges from 51 to 91% [4, 16, 17] with a pooled rate of repeat surgery of 3.4% [17].

In contrast to traditional posterior SSL fixation, the anterior access with Splentis ensures physiological positioning of the cervix, maintaining the possibility of common cancer screening and potentially reducing the risk of subsequent anterior prolapse. The first description of an anterior approach by Winkler et al. [18] demonstrated a restoration of the physiological horizontal axis of the vagina. Another study by Goldberg et al. [19] reported several advantages of using an anterior approach compared to a posterior approach, including a longer average total vaginal length, less anterior vaginal wall relaxation, and a more proximal vaginal apex. Posterior sacrospinous vaginal vault suspension leaves the vagina at a downward and posterior angle and may lead to a significant rate of cystoceles recurrence (22–25%) [20].

In contrast, unilateral sacrospinous hysteropexy may result in unphysiological horizontal positioning of the cervix by deflecting the vaginal axis posteriorly. Thus, the prevention and diagnosis of cervical or vaginal cancer may be reduced or even impossible because the cervix may no longer be accessible [17]. Additionally, deflection is considered to be the cause for the high rate of subsequent anterior prolapse [16] and the success rate of treatment of the anterior compartment is only 65.1% [17]. Furthermore, advanced stages of POP are correlated with increased failure rates of native tissue repair by SSL fixation [16].

However, the majority of women in the current investigation presented with a POP-Q stage of 3, indicating a cohort with a higher risk of failure for traditional transvaginal SSL fixation. Nevertheless, the treatment success rate remained high despite the advanced POP-Q stages in the current study.

Furthermore, the surgery time is reduced by using the vaginal route instead of the transabdominal approach [15, 21]. The mean surgery time via the vaginal route was reported to be 90 min including any native tissue repair technique [15] and 54.5 min for sacrospinous hysteropexy [17]. In contrast, the mean duration for laparoscopic mesh sacrohysteropexy was 174 min [22].

In summary, treatment success of this clinical investigation is consistent with previously reported results in the literature. Furthermore, bilateral anterior sacrospinous hysteropexy with Splentis ensures physiological positioning and mobility of the cervix, maintaining the possibility of common cancer screening.

During the perioperative course, no adverse events occurred in the current trial. Intraoperative complications, particularly visceral injuries, are rare in POP repair [17, 21]. In a large cohort trial including 507 women who were treated with laparoscopic hysteropexy, the rate of intraoperative adverse events was < 1% [23]. Thus, the current results are consistent with those in the literature.

Mesh exposure occurred in three (2.9%) patients, and only two (1.9%) patients required revision surgery which is consistent with other hysteropexy techniques including mesh placement for uterine suspension. According to a recent meta-analysis of transabdominal hysteropexy using synthetic mesh, a mean exposure rate of 3.8% was reported [15, 23]. In a prospective trial investigating vaginal versus abdominal hysteropexy techniques, the reported mesh exposure rate was 2.7 and 6.6%, respectively, without a significant difference between the groups. However, the vaginal technique included the attachment of a larger area of the mesh to the anterior vaginal wall, as previously described. Another meta-analysis comparing mesh sacrocolpopexy and vaginal native tissue repair reported a mesh complication rate of 4.2% [21]. Regarding the current investigation, the mesh exposure rates are consistent with those of other hysteropexy techniques, including mesh placement for uterine suspension.

Other adverse events, particularly those associated with transvaginal mesh-augmented POP repair for anterior prolapse [24], did not occur in the current investigation. This may be related to the mesh design and, in particular, fixation of the mesh to the uterus and sparing the vaginal wall. Only one patient (0.9%) required partial mesh excision due to dyspareunia and the formation of a granulation polyp.

Dyspareunia is one of the most commonly reported adverse events after mesh-augmented vaginal procedures [25]. However, a Cochrane meta-analysis identified only little or no difference in the dyspareunia rate between mesh-augmented and native tissue apical POP repair [26]. In the current investigation, de novo dyspareunia occurred in two subjects (1.8%), and importantly, dyspareunia, which was present at baseline, resolved after surgery in six (5.5%) patients. A recent prospective randomized trial comparing laparoscopic sacrohysteropexy with mesh and sacrospinous hysteropexy with sutures reported de novo dyspareunia in 8.1 and 13.2% of patients, respectively [27]. Considering any type of vaginal native tissue hysteropexy technique, the mean dyspareunia rate was 12.3% [14]. In comparison to unilateral sacrospinous suspension, there might be an increased risk for dyspareunia due to distortion of the vaginal configuration [17]. Therefore, Splentis might be beneficial for sexual activity due to maintenance of the physiological axis of the vagina and preservation of the uterus per se [7]. However, there was no statistically significant difference in sexual function according to the POP-Q between baseline and follow-up.

We acknowledge the current limitations of this investigation. The results of objective anatomical success are not present for the cohort. Furthermore, asymptomatic findings, such as small exposure or contractures, might have been missed because vaginal examination is often performed by the patients gynecologist. However, it should be considered the current recommendation for treatment success in POP repair focus in combined endpoints, including patient report outcome [9] and solely anatomical failure does not represent indication for retreatment. It could be demonstrated that the hymen is a relevant cut-off-point as women with prolapse beyond the hymen have more POP symptoms and are more likely to report a vaginal bulge symptom which identified this question to be crucial for defining outcome success [9]. Thus, this investigation included in particular this question at interview follow-up and additionally, the patients were also asked for any further surgery performed since last follow-up. Therefore, clinically relevant results have been collected completely. Finally, the analysis of risk factors for failure or exposure may be associated with the chance of a type II error since the number of adverse events and the anatomical failure rate were low.

## Conclusions

Bilateral anterior sacrospinous hysteropexy with Splentis offers a valid alternative for surgical correction of uterine descent while incorporating the benefits of the vaginal route. The risk-benefit ratio of Splentis appears to be favorable, and there are many theoretical benefits of this procedure that are reflected in the documented outcomes of our study.

## Supplementary Information

Below is the link to the electronic supplementary material.

Supplementary file1 (DOCX 5796 KB)

## Data Availability

The datasets used and analyses during the current study are available from the corresponding author on reasonable request.

## Abbreviations

POP: Pelvic organ prolapse
POP-Q: Pelvic organ prolapse quantification
PRO: Patient-reported outcome
SSL: Sacrospinous ligament
SUI: Stress urinary incontinence
TAS: Tissue anchoring system
