# Supplementary material for: A novel bilateral anterior sacrospinous hysteropexy technique for apical pelvic organ prolapse repair via the vaginal route: a cohort study
Source: Arch Gynecol Obstet. 2022 Mar 14;306(1):141–9. doi: 10.1007/s00404-022-06486-4 (PMC9300505; doi:10.1007/s00404-022-06486-4)
Supplement: Supplementary file 1 — Supplementary file1 (DOCX 5796 KB) [file 404_2022_6486_MOESM1_ESM.docx]

Figure S1 Schematic description of surgical steps in Splentis implantation technique

**1.** Full thickness vaginal wall dissection


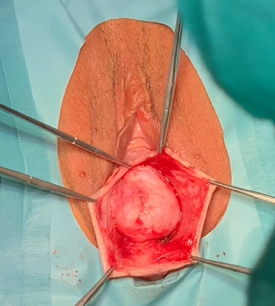


Perform a hydrodissection and subsequently a full-thickness vaginal wall dissection*

___________________________________________________

**2.** Identification of the sacrospinous ligament

Surrounding tissue of sacrospinous ligament is wiped away carefully from the ischial spine along the ligament

____________________________________________________

**3.** Implantation of the Tissue Anchoring System (TAS)

Fixation of the TAS to the sacrospinous ligament with single use instruments. Sutures attached to the anchor are utilized for fixation of the mesh ends bilaterally

_____________________________________________________________


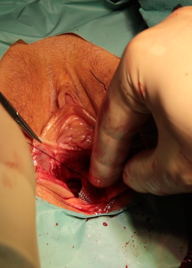

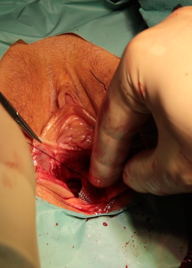
 **4.** Fixation of Splentis

Splentis is attached to the cervix. The ends of the sling-alike mesh are fixed to the sacrospinous ligament by using the suture of the TAS

__________________________________________________________

**5.** Splentis in situ

Wound closure and vaginal packing
